# Robotic modified radical hysterectomy with pelvic lymphadenectomy

**DOI:** 10.3332/ecancer.2008.55

**Published:** 2007-09-20

**Authors:** A Maggioni, L Bocciolone, M Peiretti, F Landoni, V Zanagnolo, L Minig, G Roviglione, N Colombo

**Affiliations:** Division of Gynecologic Oncology, European Institute of Oncology, Milan, Italy

## Abstract

Radical hysterectomy, the complete removal of a woman’s uterus, is usually performed via an abdominal incision that requires a 3–5 day hospital stay and a 6–8 week recovery period. Now, in a handful of hospitals around the world, new robotic technology allows doctors to perform this procedure through small incisions that require a recovery time of only one night in the hospital and a significantly shorter recovery period at home. Watch such a procedure being carried out at the European Institute of Oncology.

## Objective

To report the feasibility and safety of robotic modified radical hysterectomy and bilateral pelvic lymphadenectomy, using the four-arm da Vinci robot system, in a patient with early-stage cervical carcinoma.

## Methods

The patient is a 37-year-old woman P0G0, who was diagnosed with a severe cervical dysplasia on a pap smear during a regular follow-up check-up visit. She underwent a biopsy that revealed a grade three invasive cervical squamous carcinoma, and she was referred to our institute. The computerized tomography of the abdomen and pelvis was negative for metastatic disease, and MRI of the pelvis revealed a cervical lesion of 16 × 12 mm. She was therefore offered a radical hysterectomy, using the da Vinci robotic set. She signed an informed consent form, and the risks and benefits of open surgery, versus a minimally invasive procedure, were discussed.

**Figure:**
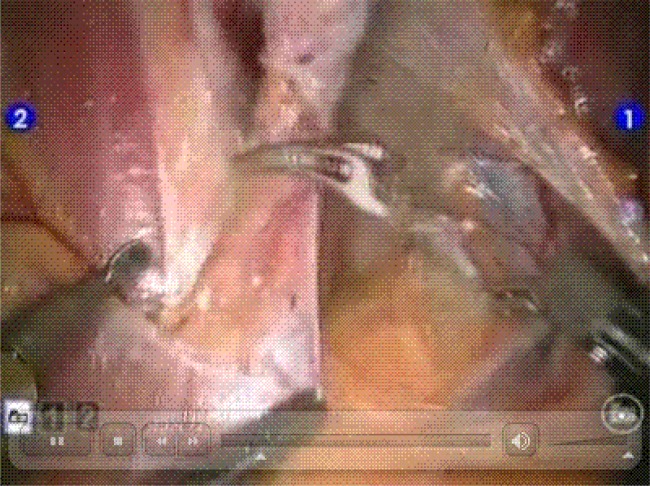
http://www.ecancermedicalscience.com/view-article.asp?doi=10.3332/ecancer.2008.55

## Results

The overall operative time was two hours and 20 minutes, with only two minutes for docking of the robotic arms. Estimated blood loss was <50 cc. There were no intra-operative or postoperative complications. Patient removed Foley catheter on post-operative day three, and she went home the same day with intermittent self-catheterization. Pathological findings revealed a squamous cell carcinoma G3 with a depth of stromal invasion of 6 mm and horizontal extension of 12 mm. No lymph vascular spaces involvements were noted. Bilateral parametria and vaginal margins were free of the disease. Twenty-six negative pelvic lymph nodes were removed.

## Conclusion

Modified radical hysterectomy with bilateral pelvic lymphadenectomy using the four-armed da Vinci Robot seems to be a safe and feasible procedure. Surgeons can clearly benefit from better dexterity precision and visualization with the three-dimensional imaging than conventional laparoscopy. Limitations such as the absence of tactile feedback and high costs will need to be addressed from further controlled studies.

